# Involvement of Hepcidin in Cognitive Damage Induced by Chronic Intermittent Hypoxia in Mice

**DOI:** 10.1155/2021/8520967

**Published:** 2021-08-04

**Authors:** Ya-Shuo Zhao, Miao Tan, Ji-Xian Song, Ji-Ren An, Xin-Yue Yang, Wen-Ya Li, Ya-Jing Guo, En-Sheng Ji

**Affiliations:** ^1^Department of Physiology, Institute of Basic Medicine, Hebei University of Chinese Medicine, Shijiazhuang 050200, China; ^2^Hebei Technology Innovation Center of TCM Formula Preparations, Shijiazhuang 050200, China

## Abstract

Obstructive sleep apnea (OSA) patients exhibit different degrees of cognitive impairment, which is related to the activation of reactive oxygen species (ROS) production by chronic intermittent hypoxia (CIH) and the deposition of iron in the brain. As a central regulator of iron homeostasis, whether hepcidin is involved in OSA-induced cognitive impairment has not been clarified. In order to simulate OSA, we established the mouse model by reducing the percentage of inspired O_2_ (FiO_2_) from 21% to 5%, 20 times/h for 8 h/day. We found hepcidin was rising during CIH, along with increasing iron levels and neuron loss. Then, we constructed a mouse with astrocyte-specific knockdown hepcidin gene (sh*Hamp*). During CIH exposure, the sh*Hamp* mice showed a lower level of total iron and neuronal iron in the hippocampus, via stabilizing ferroportin 1 (FPN1) and decreasing L-ferritin (FTL) levels, when compared with wild-type (WT) mice. Furthermore, the sh*Hamp* mice showed a decrease of ROS by downregulating the elevated NADPH oxidase (NOX2) and 4-hydroxynonenal (4-HNE) levels mediated by CIH. In addition, the sh*Hamp* mice presented improved cognitive deficit by improving synaptic plasticity and BDNF expression in the hippocampus when subjected to CIH. Therefore, our data revealed that highly expressed hepcidin might promote the degradation of FPN1, resulting in neuronal iron deposition, oxidative stress damage, reduced synaptic plasticity, and impaired cognitive performance during CIH exposure.

## 1. Introduction

Obstructive sleep apnea (OSA) is a prevalent sleep breathing disorder. Clinically, the OSA patient is characterized by the repeated cycle of the upper airway obstruction and arousal during sleep, which leads to chronic intermittent hypoxia (CIH), hypercapnia, hypoxemia, and sleep fragmentation, resulting in daytime sleepiness and memory decline and degradation of reactivity [1]. Epidemiological study has demonstrated that the number of OSA patients worldwide has exceeded 9.36 million, and it is becoming a serious global problem [2]. OSA has been identified as an early risk factor for developing neurodegenerative disease, such as Alzheimer's disease (AD) [1, 3]. Clinical studies have shown that OSA could accelerate the progression of AD by increasing the formation and accumulation of A*β*_42_ in the cerebrospinal fluid (CSF) [4, 5]. OSA is often overlooked by patients in the early stage. Therefore, it is particularly necessary to explore further mechanisms involved in the pathogenesis and conduct appropriate intervention and treatment.

Similar to ischemia-reperfusion, the CIH-induced hypoxia/reoxygenation is the most important pathological feature of OSA, which could cause the formation of a large number of reactive oxygen species (ROS) [6]. The levels of oxidative stress markers in serum of OSA patients are increased, which is related to the severity of OSA [7]. The elevated ROS could cause endoplasmic reticulum stress, induce the changes of mitochondrial membrane potential, and activate the MAPK signaling pathway, resulting in neuronal cell apoptosis in the hippocampus of rodents [8]. The excessive ROS could also induce lipid peroxidation in the hippocampus to promote structural damage of neurons, increase the synaptic space, and reduce the long-term enhancement effect involved in memory impairment [9, 10].

Hepcidin, predominantly produced and secreted by the liver, is the master regulator of systemic iron availability and plays a role by controlling ferroportin1 (FPN1), the sole iron export protein [11]. Hepcidin has also been shown to be expressed in the brain of glial cells, predominantly in astrocytes, rather than mature neurons [12, 13]. Hepcidin appears to control iron entry from plasma into the brain and the transfer in different neural cells [13]. Circulating iron is transported via Tf/TfR1 to cross the blood-brain barrier (BBB). Subsequently, the complexity of Fe-Tf/TfR1 undergoes endocytosis, which releases iron into intracellular by divalent metal transporter 1 (DMT1). The cytoplasmic iron is used to produce heme and is excreted by via FPN1 or stored in ferritin [13–15]. The excess free iron could generate free radicals by Fenton reaction, resulting in oxidative damage in neural cells, which has demonstrated in AD [16], Parkinson's disease [17], and ischemic stroke [18, 19].

Clinically, OSA patients exhibit a high level of hepcidin, lower iron, and transferrin saturation (TSA) in serum [20–22]. Correlation analysis clarifies that serum hepcidin level is positively correlated with sleep-disordered breathing index and disease severity [20, 23]. Our previous study revealed that CIH exposure could induce the mobilization and absorption of iron, leading to iron deposition in the hippocampal CA1, CA3, and dentate gyrus (DG) [24]. However, whether hepcidin is involved in CIH-induced cognitive deficit remains unclear. Therefore, we established the mouse model of CIH to simulate OSA and explored the hepcidin expression at different CIH simulating times. In addition, we constructed genotypic mice with specific knockdown of hepcidin in astrocytes (sh*Hamp*), so as to study the relationship of hepcidin and CIH-induced neurocognitive impairment.

## 2. Materials and Methods

### 2.1. Experimental Animals and Grouping

The SPF C57BL/6N mice (male, 20 g ± 2 g) were purchased from Beijing Vital River Laboratory Animal Technology Co., Ltd. (Beijing, China). All mice were adapted to their living conditions for at least 7 days before the experiment. All animal experimental procedures were carried out in strict accordance with the National Institutes of Health *Guide for the Care and Use of Laboratory Animals* and approved by the Animal Care and Use Committee of Medical Ethics of Hebei University of Chinese Medicine (No. DWLL2018006).

**Figure 1:**
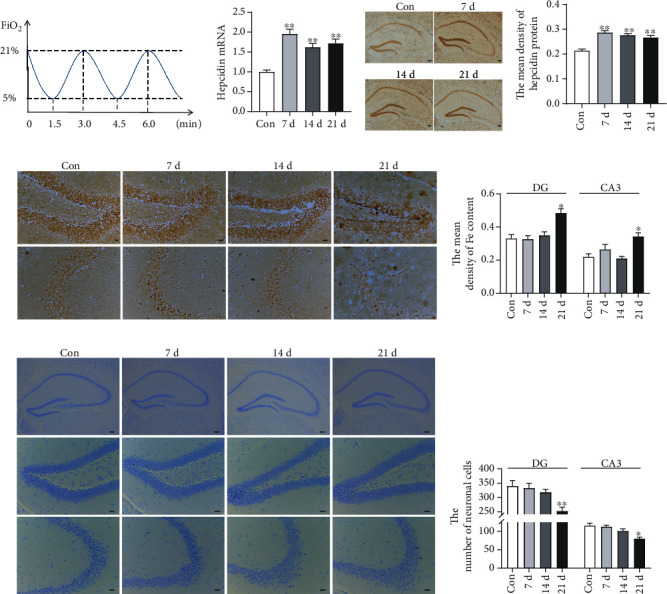
The elevated hepcidin and Fe content in the hippocampus with different exposed times. (a) The flow chart of CIH exposure procedure. (b) Hepcidin mRNA level determined by q-PCR in the hippocampus of mice subjected to CIH for 7 d, 14 d, and 21 d (*n* = 6). (c) The hepcidin protein expression detected by immunohistochemistry (scale bar = 100 *μ*m). (d) The mean density of hepcidin protein expression in the whole hippocampus as shown in panel (c) (*n* = 3). (e) Perls' staining of the dentate gyrus (DG) and hippocampal CA3 (scale bar = 25 *μ*m). (f) The mean density of Fe content as shown in panel (e) (*n* = 3). (g) The Nissl staining of the hippocampus subjected to CIH for 7 d, 14 d, and 21 d (scale bar = 100 or 25 *μ*m). (h) The number of neuronal cells in the dentate gyrus (DG) and hippocampal CA3 as shown in panel (g) (*n* = 3). The data are shown as the means ± SEM. ^∗^*p* < 0.05 and ^∗∗^*p* < 0.01 vs. Con.

We established a murine CIH model to simulate OSA. The mice were placed in a chamber in which the fraction of inspired oxygen (FiO_2_) was decreased from 21% to 5% and then gradually returned to 21%. The exposure cycle was repeated every 3 min, 20 times/h for 8 h/day (Figure 1(a)). The mice in the control group (Con) received normal air (21% O_2_) in the identical chambers. The C57BL/6N mice (*n* = 24) were randomly assigned to the four CIH exposure groups, and there was a washout period of 7 days between CIH exposures.

**Figure 2:**
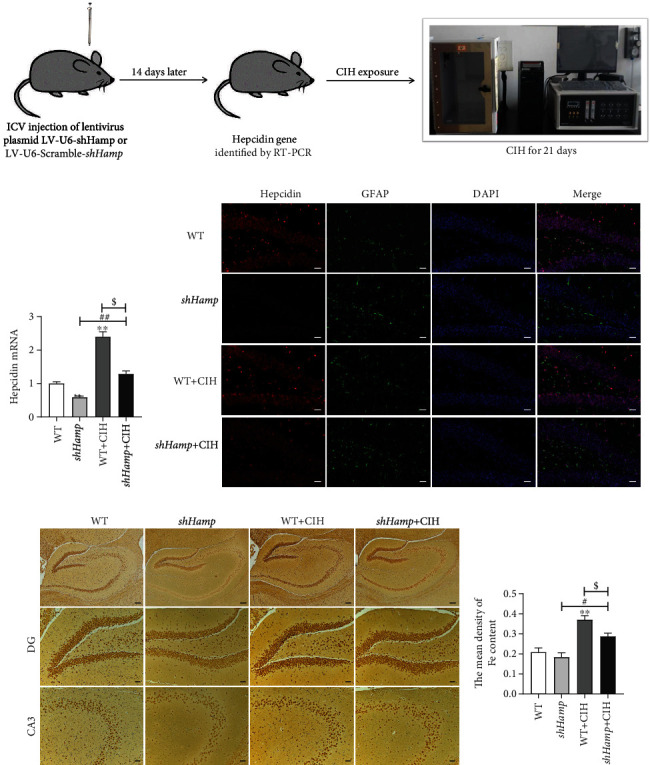
The preparation and identification of sh*Hamp* mice and iron level. (a) The prepared pattern diagram of sh*Hamp* mice of astrocyte-specific knockdown hepcidin. (b) Hepcidin mRNA level determined by q-PCR in the hippocampus of WT and sh*Hamp* mice exposed to CIH for 21 days (*n* = 6). (c) Double immunofluorescence staining in the hippocampal dentate gyrus (DG). The sections were labeled for hepcidin (red), GFAP (green), and DAPI (blue) (scale bar = 25 *μ*m, *n* = 3). (d) Perls' staining of the dentate gyrus (DG) and hippocampal CA3 (scale bar = 100 *μ*m or 25 *μ*m). (e) The mean density of Fe content as shown in panel (d) (*n* = 3). The data are shown as the means ± SEM. ^∗^*p* < 0.05 and ^∗∗^*p* < 0.01 vs. WT. #*p* < 0.05 vs. sh*Hamp*. ^$^*p* < 0.05 vs. WT+CIH.

Next, we prepared the wild-type (WT) and sh*Hamp* mice (*n* = 10 for each group) and exposed to CIH for 21 days. Firstly, we constructed a lentivirus plasmid LV-U6-sh*Hamp* with the astrocyte promoters followed by *Hamp* gene shRNA sequence “ACCGGGCAGACATTGCGATACCAATTCTCGAGAATTGGTATCGCAATGTCTGCTTTTTGAATTC” (Figure S1A). The 4 *μ*l LV-U6-sh*Hamp* plasmid (virus titer ≥ 1 × 10^8^ TU/ml) was injected into the lateral ventricle of mice. The WT mice were given the same volume LV-U6-Scramble-sh*Hamp* with the astrocyte promoters followed by *Hamp* gene control shRNA sequence. Two weeks later, the hepcidin gene was identified by RT-PCR. When the hepcidin gene expression was decreased off about 50%, the sh*Hamp* mice were considered to be successful. Then, the WT and sh*Hamp*, respectively, were exposed to CIH for 21 days to evaluate the changes of behavioristics, pathology, and molecular biology (Figure 2(a)).

### 2.2. Reagents and Antibodies

Reagents include lentivirus plasmid (Cyagen Biosciences), potassium ferrocyanide (Sigma-Aldrich), DAPI (2 mg/ml, Servicebio), DHE (Cayman Chemical), protease inhibitors (Thermo Fisher), and phosphatase inhibitors (Servicebio). The TUNEL kit was purchased from Vazyme Biotech. The BCA protein assay kit was purchased from CoWin Biosciences. RNA extraction kit was purchased from Tiangen Biotech.

Antibodies used were as follows: hepcidin (1 : 100, Affinity), TfR1 (1 : 10000, Invitrogen), FPN1 (1 : 5000 for WB, 1 : 200 for IF, Alpha Diagnostic International), FTL (1 : 5000 for WB, 1 : 200 for IF, Abcam), GFAP (1 : 100, Servicebio), NeuN (1 : 200, Abcam), Bcl-2 (1 : 2000, ImmunoWay), Bax (1 : 1000, Servicebio), p-JNK (1 : 1000, Cell Signaling Technology), JNK (1 : 1000, Arigo Biolaboratories), NOX2 (1 : 2000, GeneTex), 4-HNE (1 : 200 for IF, 1 : 1000 for WB, Arigo Biolaboratories), BDNF (1 : 1000, Servicebio), *β*-actin (1 : 1000, Cwbiotech), and *β*-tubulin (1 : 1000, Servicebio).

### 2.3. Morris Water Maze

**Figure 3:**
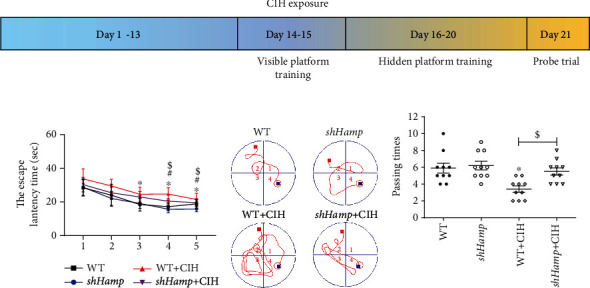
Morris water maze of the WT and sh*Hamp* mice treated with CIH for 21 days. (a) The flow chart of Morris water maze. (b) The escape latency time was performed on days 16-20. (c) The escape route distance on day 20. (d) The number of passing times of probe trial on day 21. The data are shown as the means ± SEM (*n* = 10). ^∗^*p* < 0.05 vs. WT. ^#^*p* < 0.05 vs. sh*Hamp*. ^$^*p* < 0.05 vs. WT+CIH.

The Morris water maze (MWM) was used to assess memory function as previously described [16]. As shown in Figure 3(a), in the first two days, the visible platform (the platform above the water surface) was used for training. The hidden platform (the platform below the water surface) was used for training in the next five days. Each mouse was released into a quadrant facing the wall of the water tank. The time spent to find the hidden platform was referred as latency time, and latency route and distance were also recorded. The latency time and route from the opposite side of the platform to the platform are counted and calculated. On the 6th day, the hidden platform was removed for the probe trial, and then, the times crossing the platform of original position were recorded within 2 min.

### 2.4. Perls' Staining

The iron distribution in the hippocampus was evaluated through Perls' staining. After dewaxed, the sections were dipped in PBS (0.01 M) and treated with 3% H_2_O_2_ for 20 min to remove endogenous peroxidase. The sections were immersed in fresh Perls' solution containing 1% potassium ferrocyanide and 1% hydrochloric acid for 10 h. After washed thoroughly in PBS, the sections were strengthened with DAB kits, dehydrated, and finally covered with neutral balsam. The mean density was calculated by Image-Pro Plus 6.0 software.

### 2.5. TUNEL

The TUNEL staining was in accordance to the instruction as previously described [24]. The frozen sections were cleaned with PBS and incubated with equilibration buffer (EB) to remove endogenous peroxidase. The newly configured reaction mixture of Bright Green Labeling Mix and Recombinant TdT Enzyme was added into the sections at 37°C for 1 h. After washing, the sections were incubated with DAPI at room temperature. The antifluorescence quencher was used to seal the sections. FITC and DAPI fluorescence were detected at 488 nm and 460 nm, respectively. The number of total apoptotic cells was calculated by Photoshop CS 5.0 software.

### 2.6. DHE Staining

The dihydroethidium (DHE) staining was adopted to measure the ROS level in the hippocampus. The frozen sections were cleaned with PBS and added self-fluorescence quenching agent for 5 min, then incubated with 5 *μ*M of DHE at 37°C for 30 min in dark. The sections were rewashed and sealed with an antifluorescence quencher. The red fluorescence was detected at 549 nm. The mean density was calculated by Image-Pro Plus 6.0 software.

### 2.7. Immunohistochemistry

The frozen sections were washed with PBS and incubated with 3% H_2_O_2_ to block endogenous peroxidase. The antigen retrieval was applied with the boiling citrate (10 mM, pH 6.0) method. After the washing stage, the sections were incubated with 10% goat serum for 60 min at room temperature (RT).

Immunohistochemistry was performed as follows. The brain sections were incubated with primary antibodies hepcidin and 4-HNE overnight at 4°C. On the following day, the slides were incubated with the HRP-conjugated second antibody at 37°C for 60 min. Subsequently, DAB staining, dehydration, hyalinization, and mounting were performed successively.

The double immunofluorescence was performed as follows. The brain sections were incubated with the mouse anti-GFAP or NeuN antibody and rabbit antihepcidin or FPN1 or FTL antibody overnight at 4°C. On the following day, the secondary antibodies, DyLight 549 goat anti-rabbit IgG and DyLight 488 goat anti-mouse IgG, were added to incubate the brain sections and were incubated at 37°C for 60 min. After washing, the sections were sealed with an antifluorescence quencher and visualized with a fluorescence microscope.

### 2.8. Transmission Electron Microscopy

The ultrastructural change of synapses was visualized using transmission electron microscopy (TEM). After deep anesthesia, the hippocampal dentate gyrus (DG) slices were removed and fixed using osmium tetroxide. Then, the sample was prepared according to the standard procedures. The ultrathin sections (80 nm) were collected and stained with uranyl acetate and lead nitrate. The sections were observed under a Hitachi HT7800/HT7700 electron microscope.

### 2.9. Western Blot Analysis

Western blot was performed to detect protein expression. First, the brain tissues were homogenized in 4°C RIPA lysis buffer, which contained protease inhibitors and phosphatase inhibitors. After centrifugation, the supernatants were collected, and the total protein concentrations were determined with BCA protein assay kit. The proteins were separated by SDS-PAGE and transferred into PVDF membranes. The membranes were blocked with 5% skim milk powder for 2 h at RT and incubated with primary antibodies: TfR1, FPN1, FTL, NOX2, 4-HNE, Bcl-2, Bax, p-JNK, JNK, BDNF, *β*-actin, and *β*-tubulin at 4°C overnight. On the following day, the blots were washed with TBST and incubated with HRP-conjugated secondary antibodies for 1 h at RT. The immunoreactive protein bands were imaged using chemiluminescence method. The mean density of the bands was calculated by ImageJ software.

### 2.10. PCR

The total RNA was extracted from the hippocampus using RNA extraction kit following the manufacturer's instructions. 1 *μ*g total RNA was reversely transcribed into cDNA. RT-PCR was used to identify the expression of hepcidin mRNA (primer 1) of sh*Hamp* mouse. The quantitated hepcidin mRNA (primer 2) in WT mouse was used as q-PCR with the Bio-Rad CFX Connect system. The primer sequences used were as follows: *β*-actin: forward: AGACATTGCGATACCAATGCA, reverse: GCAACAGATACCACACTGGGAA; hepcidin primer 1: forward: TATCTCCGGCAACAGACGAG, reverse: TGTCTCGCTTCCTTCGCTTC; and hepcidin primer 2: forward: AGGCCCAGAGCAAGAGAGGTA, reverse: TCTCCATGTCGTCCCAGTTG.

### 2.11. Statistical Analyses

The results are presented as the mean ± SEM. The statistical analysis was performed using one-way ANOVA followed by the LSD post hoc test or *t*-test. Two-way ANOVA was used to analyze the results of the behavioral tests. The significance level was regarded as *p* < 0.05.

## 3. Results

### 3.1. Hepcidin Expression in the Hippocampus with Different Exposed Times

The expression of hepcidin gene and protein in the hippocampus was measured with different exposed times. As shown in Figure 1(b), hepcidin mRNA level was increased after CIH exposure, and the rise persisted till to the day 21 of the experiment. Consistent with gene expression, immunohistochemical analysis showed that the expression of hepcidin protein in the hippocampus tissue was increased (Figures 1(c) and 1(d)). Furthermore, the distributed iron in the hippocampus was evaluated by Perls' staining. The images presented that the exposure of CIH could significantly lead to the iron deposition in the mouse hippocampus, especially lasted to 21 days (Figures 1(e) and 1(f)). In addition, Nissl staining revealed that the Nissl body staining became shallow and small when CIH was exposed to 21 days, indicating the loss of neurons (Figures 1(g) and 1(h)). Therefore, we chose 21 days as the time point of CIH exposure in the subsequent experiment. These results suggested that the high level of hepcidin might contribute to iron deposition and neuron loss in the hippocampus.

### 3.2. Astrocyte-Specific Knockdown Hepcidin Decreased the Elevation of Iron Induced by CIH

To further characterize the role of hepcidin in CIH-induced cognitive deficit, the mice of astrocyte-specific knockdown hepcidin were established. Firstly, we compared hepcidin gene and protein expression between sh*Hamp* and WT mice. RT-PCR revealed that hepcidin mRNA gene was decreased off 54% after lateral ventricular injection of LV-U6-sh*Hamp* (Figure S1B). Therefore, we chose 14 days as the time point of sh*Hamp* mouse preparation.

Then, hepcidin mRNA levels were determined by q-PCR in the hippocampus of sh*Hamp* mice exposed to CIH for 21 days. Compared to the WT group, hepcidin mRNA levels were lower in the sh*Hamp* group when exposed to normal air or CIH (Figure 2(b)). Meanwhile, the double immunofluorescence showed repression of hepcidin expression secreted by astrocyte in sh*Hamp* mice; the hepcidin protein level was still lower than the normal even though it was elevated by CIH exposure (Figure 2(c)). Compared with the WT mice, the iron-positive cells were decreased in the hippocampus of sh*Hamp* mice during CIH exposure (Figures 2(d) and 2(e)).

**Figure 4:**
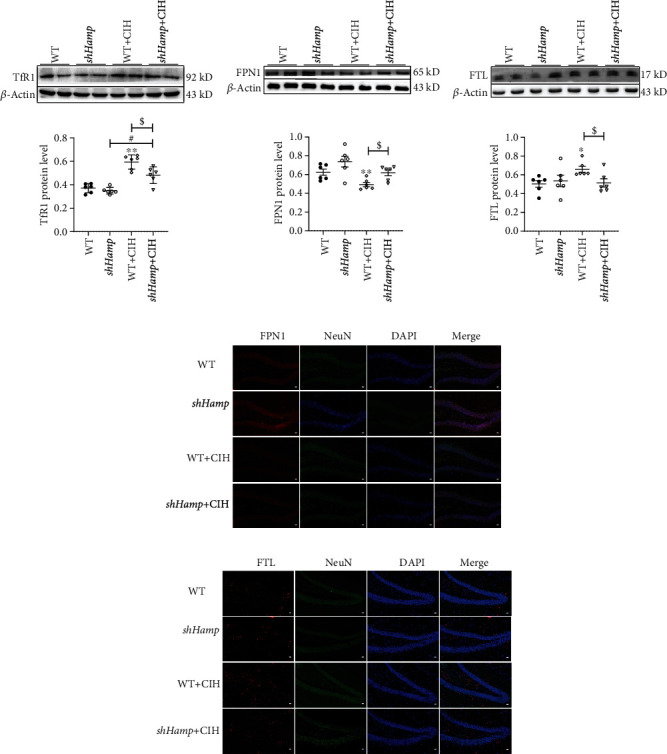
The expression of proteins related to iron metabolism in the hippocampus tissue and neurons. (a–c) The expression of TfR1, FPN1, and FTL proteins measured by western blot (*n* = 6). (d, e) Double immunofluorescence staining in the hippocampal dentate gyrus (DG). The sections were labeled for FPN1 or FTL (red), NeuN (green), and DAPI (blue) (scale bar = 25 *μ*m, *n* = 3). The data are shown as the means ± SEM. ^∗^*p* < 0.05 and ^∗∗^*p* < 0.01 vs. WT. ^#^*p* < 0.05 vs. sh*Hamp*. ^$^*p* < 0.05 vs. WT+CIH.

However, how did the iron levels rise? For this purpose, we detected the related proteins involved in iron metabolism. The western blot results showed that the expression of TfR1, iron intake of protein, was increased when subjected to CIH in the WT and sh*Hamp* mice (Figure 4(a)). During CIH exposure, the sh*Hamp* exhibited a lower level of TfR1 compared with WT mice (Figure 4(a)). Hepcidin regulated iron levels in neural cells mainly by binding to the iron-releasing protein FPN1. We further found that the expression of FPN1 protein elevated in the hippocampal tissues and neuron of sh*Hamp* mice (Figures 4(b) and 4(d)). Compared with the WT mice, the FPN1 protein levels in sh*Hamp* mice increased after CIH exposure (Figure 4(b)). Besides, we examined the FTL protein expression in the hippocampal and neurons to indirectly determine the iron levels. Similar to our previous studies, the FTL protein levels rose in the hippocampal tissues and neuron during CIH exposure (Figures 4(c) and 4(e)). The sh*Hamp* mice showed a lower level of FTL when exposed to CIH (Figure 4(c)). These results suggest that the knockdown of hepcidin might decrease the CIH-induced iron deposition, especially in neurons.

### 3.3. Decreased ROS Levels in sh*Hamp* Mice Attenuated CIH-Induced Oxidative Stress

**Figure 5:**
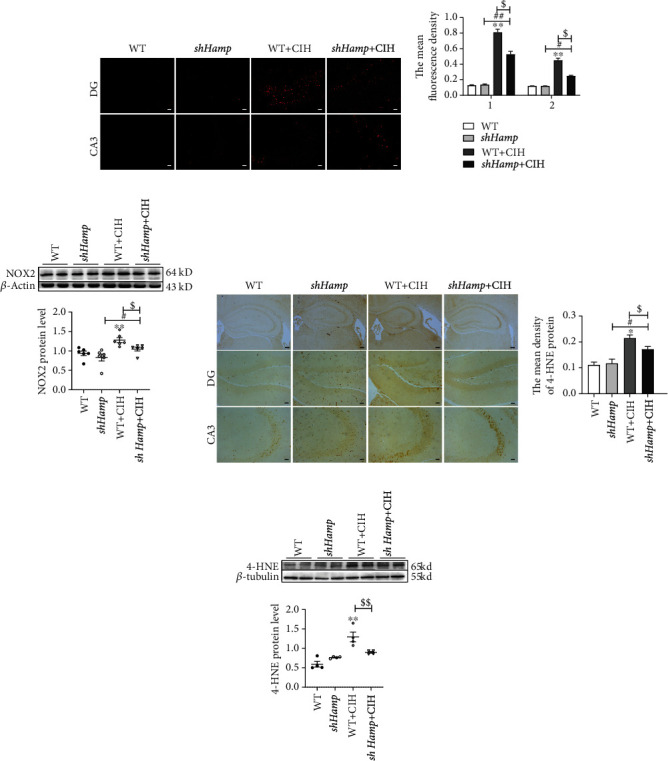
The oxidative stress levels in the WT and sh*Hamp* mice treated with CIH. (a) The DHE staining of the hippocampal dentate gyrus (DG) and hippocampal CA3 (scale bar = 25 *μ*m, *n* = 3). (b) The mean fluorescence intensity as shown in panel (a). (c) The expression and statistics of NOX2 protein (*n* = 6). (d) The immunohistochemical staining of 4-HNE protein in the hippocampal dentate gyrus (DG) and hippocampal CA3 (scale bar = 25 *μ*m, *n* = 3). (e) The mean density of 4-HNE protein as shown in panel (d). (f) The expression and statistics of 4-HNE protein measured by western blot (*n* = 4). The data are shown as the means ± SEM. ^∗^*p* < 0.05 and ^∗∗^*p* < 0.01 vs. WT. ^#^*p* < 0.05 and ^##^*p* < 0.01 vs. sh*Hamp*. ^$^*p* < 0.05 and ^$$^*p* < 0.01 vs. WT+CIH.

An excess of iron contributes to ROS production by Fenton reaction. Therefore, we detected the ROS levels and oxidative damage induced by CIH. We firstly found with the help of DHE probes that compared with WT mice, the increased mean fluorescence intensity in sh*Hamp* mice was significantly reduced during CIH exposure (Figures 5(a) and 5(b)). NADPH oxidases (NOXs) could participate in ROS production in the mitochondria. Compared with WT mice, western blot results revealed that the high level of NOX2 proteins was receded in the sh*Hamp* mice when subjected to CIH (Figure 5(c)). 4-Hydroxynonenal (4-HNE), one of lipid peroxidation products, was increased in the WT mice with CIH exposure (Figures 5(d)–5(f)). However, the higher 4-HNE level was weakened in the mice of the sh*Hamp*+CIH group (Figures 5(d)–5(f)). These results reveal that hepcidin deficiency could reduce CIH-induced oxidative stress damage.

### 3.4. Effects of Hepcidin Deficiency on the Apoptosis in the Hippocampus

**Figure 6:**
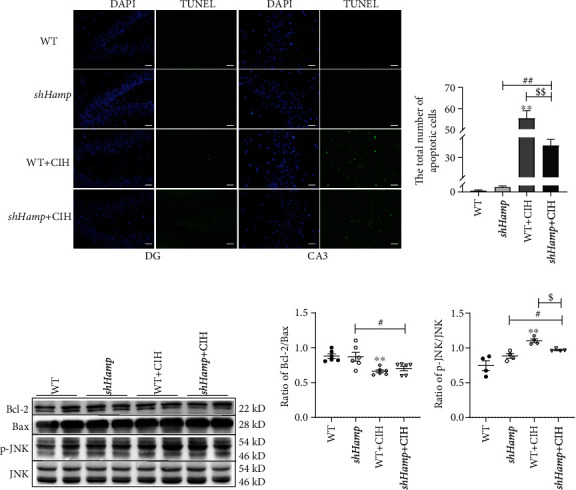
The apoptosis in the hippocampus of WT and sh*Hamp* mice exposed to CIH. (a) The TUNEL staining of the hippocampal dentate gyrus (DG) and hippocampal CA3 (scale bar = 25 *μ*m, *n* = 3). (b) The total number of apoptotic bodies as shown in panel (a). (c–e) The ratio of Bcl-2/Bax (*n* = 6) and p-JNK/JNK (*n* = 4) proteins. The data are shown as the means ± SEM. ^∗∗^*p* < 0.01 vs. WT. ^#^*p* < 0.05 and ^##^*p* < 0.01 vs. sh*Hamp*. ^$^*p* < 0.05 and ^$$^*p* < 0.01 vs. WT+CIH.

Oxidative stress is an initial factor of neuronal apoptosis when subjected to CIH exposure. We used TUNEL staining to observe the loss of neurons in the hippocampus induced by CIH. As shown in Figures 6(a) and 6(b), a considerable number of apoptosis bodies existed in the hippocampus of WT mice subjected to CIH. However, the number of apoptosis bodies of the sh*Hamp*+CIH group was lower than that of the WT+CIH group. Meanwhile, we found that the decreased ratio of Bcl-2/Bax induced by CIH was increased in the sh*Hamp* mice (Figures 6(c) and 6(d)). The ratio of p-JNK/JNK showed elevation in the WT and sh*Hamp* mice exposed to CIH, respectively (Figures 6(c) and 6(e)). Compared with the WT+CIH group, the ratio of p-JNK/JNK was decreased in the sh*Hamp*+CIH group. These results suggest that hepcidin deficiency reduces apoptosis induced by CIH exposure.

### 3.5. Synaptic Plasticity and Recognition Memory Improvement in sh*Hamp* Mice Exposed to CIH

**Figure 7:**
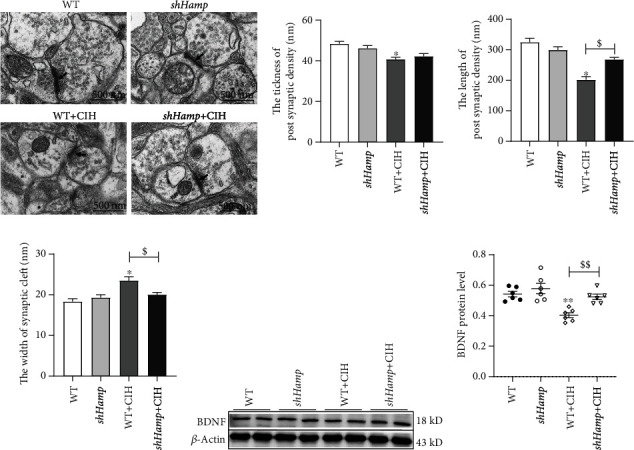
The change of synaptic structure and BDNF expression in the WT and sh*Hamp* mice exposed to CIH. (a) The TEM of synaptic structure of the mouse hippocampus in four different groups (*n* = 3). (b) The thickness of postsynaptic density. (c) The length of postsynaptic density. (d) The width of synaptic cleft. (e, f) The expression of BDNF protein detected by western blot (*n* = 6). The data are shown as the means ± SEM. ^∗^*p* < 0.05 and ^∗∗^*p* < 0.01 vs. WT. ^$^*p* < 0.05 and ^$$^*p* < 0.01 vs. WT+CIH.

Studies have been reported that the decrease of synaptic plasticity was related to memory loss induced by CIH. We try to clarify hepcidin might be involved in memory loss during CIH exposure. As shown in Figure 7, the TEM images showed that the thickness and length of postsynaptic density (PSD) were smaller and the width of synaptic cleft was wider in the CIH group compared to that in the WT group. This indicated that the synaptic structure was damaged induced by CIH exposure. Furthermore, the injury of synapse morphology was partially rescued in sh*Hamp* mice when compared with the WT+CIH group (Figures 7(a), 7(c), and 7(d)). In addition, the expression of BDNF was significantly reduced in the WT mice treated with CIH (Figure 7(e)). The BDNF level was increased in the sh*Hamp*+CIH group compared with that in the WT+CIH group. These results showed that the synaptic plasticity was improved in sh*Hamp* mice to resist to CIH-induced neurological impairment.

Furthermore, we studied the behavioral changes in WT and sh*Hamp* mice during CIH. The MWM was carried out as shown in Figure 3(a). After exposure to CIH, the escape latency time on days 18-20 was increased in the WT+CIH group (Figure 3(b)). Compared with the WT+CIH group, the escape latency time on days 19-20 was decreased in the sh*Hamp*+CIH group. As shown in Figure 3(c), the escape route distance on day 20 was decreased in the sh*Hamp*+CIH group compared with that in the WT+CIH group. On day 21, the CIH mice exhibited a decrease of number of passing times during the probe trial (Figure 3(d)). The decreased platform crosses were elevated in the sh*Hamp* mice. These results indicate that the gene loss of hepcidin could improve the cognitive dysfunction when exposed to CIH.

## 4. Discussion

OSA is an independent risk factor for the development of cognitive impairment. Our previous studies have proved that it is mainly attributed to oxidative stress injury and iron deposition in the hippocampus of mice exposed to CIH. However, the mechanisms underlying how the different neural cells are involved in regulating iron remain largely unknown. In this study, we revealed an underappreciated role of hepcidin in the cognitive impairment induced by CIH. During three weeks of CIH exposure, with 8 hours per day of 5% O_2_ saturation, hepcidin was induced in the hippocampus. The high expression of hepcidin secreted by astrocyte was associated with excessive iron in neurons in the CIH mouse model. Specific deficiency of hepcidin gene could alleviate the toxic damage of iron induced by CIH.

Hepcidin plays a fundamental role in maintaining systemic iron by inhibiting iron absorption from the duodenum and releasing from macrophages via degradation of FPN1 [25, 26]. The abnormally elevated expression of hepcidin is associated with various chronic conditions, which may lead to iron excess [11]. However, deletion of hepcidin gene is also a key factor to induce iron overload, which shares the similar pathological phenotypes as hereditary hemochromatosis (HH) [11, 27, 28]. Evidence from animal studies suggests that young *Hamp* knockout mice present dramatically systemic iron overload reflected in elevated serum and hepatic iron, owing to the failure of hepcidin-FPN1 axis [28]. The iron deposition of *Hamp* knockout mice presents a time-dependent trend in peripheral organs [28], rather than in the brain [29]. It has been reported that inhibition of hepcidin expression in the brain could weaken iron accumulation and oxidative injury in the rodent model of ischemia-reperfusion [18] or intracerebral hemorrhage [29], which indicated that the brain iron metabolism was readily regulated by the local hepcidin [25].

As the most abundant cell type within the CNS, astrocytes are the main type of glial cells and play essential roles in iron transport of the brain, most particularly in maintaining the iron transport in neuronal cells [30]. Astrocytes are also the main glia cells which produce and secrete hepcidin [13, 30]. Studies have demonstrated that CIH exposure could further induce the activation of astrocytes in APP/PS1 mice and exacerbate the pathogenesis of disease [3]. Coincidentally, results of the present study indicated the increased astrocyte activation during CIH exposure, and the expression level of hepcidin also increased concomitantly (Figure 1). Some studies have indicated that high expression of hepcidin is able to promote the degradation of FPN1 and iron deposition in neurons in the ischemic brain or LPS stimulus [18, 31]. Our previous study found a decrease of FPN1 protein in the hippocampus when exposed to CIH for 21 days [24]. In the brain, FPN1 is more expressed in the cell membranes of neurons, which means that the neuron is vulnerable to the toxicity of iron [15]. Therefore, lower level of FPN1 and higher level of FTL indicated an intracellular iron accumulation status of neuron exposing to CIH (Figure 4). The data indicates that unbalance of hepcidin-FPN1 might be the main contributor to the iron overload in the hippocampus of mice exposed to CIH.

In addition, the iron-responsive element (IRE)/iron regulatory proteins (IRPs) are also important posttranscriptionally regulatory switch during different iron states [32]. FTL and TfR1 mRNA contain IRE in their 5′ UTR and 3′ UTR, respectively [32]. During CIH exposure, iron mobilization plays an indispensable role in meeting the oxygen and energy demand of the brain, and thus, the expression of TfR1 is upregulated to improve iron uptake by promoting the combination of IRE and IRPs [24]. The activity of IRE-binding IRPs is diminished in states of iron overload and thereby plays a role in inhibiting TfR1 expression and promoting FTL expression [33, 34]. Similarly, the expression of TfR1 was consistent with the iron level in the sh*Hamp*+CIH group compared to that in the WT+CIH group. Concordantly, we found a decreased expression of FTL in the sh*Hamp*+CIH group, which may be attributed to the decrease in iron levels without the need for excessive FTL for binding. These results inferred that the hepcidin deficiency influenced iron uptake and decreased brain iron overload, thereby protecting against CIH-induced injury.

However, the role of hepcidin in the development of cognitive impairment-related diseases remains controversial. The AD mice show a decrease of hepcidin in the CSF or hippocampus [16, 35]. If the hepcidin is overexpressed in astrocytes, amyloid-*β* induced the cognitive impairment and toxic iron damage in neurons could be improved [34]. Nevertheless, the hepcidin has a reverse expression level in the stroke animal models. Adding hepcidin could aggravate brain injury and iron overload in the rats subjected to subarachnoid hemorrhage [36]. Specific inhibition of the expression of hepcidin has been demonstrated to attenuate neurologic deficit and brain iron deposition in the rodent models of subarachnoid hemorrhage [36], ischemic stroke [18, 19], and intracerebral hemorrhage [29]. In our study, inhibition of hepcidin gene also improved the cognition ability of CIH mice. The reason may be attributed rather to the similar pathological characteristics of CIH model to ischemia-reperfusion, which mainly manifests as repeated hypoxia and reoxygenation recycling [6].

CIH exposure in OSA leads to increased ROS production; excessive iron generates hydroxyl radicals via the Fenton reaction and induces the overproduction of ROS, resulting in neuronal damage consequently [37–39]. In vitro experiment has confirmed that there is a positive correlation between iron level and ROS content in neurons [39]. Likewise, lower level of iron and ROS has been demonstrated in the sh*Hamp* mice subjected to CIH exposure. NADPH oxidase (NOX) enzymes could transport electrons across the plasma membrane and participate in the production of ROS in the mitochondria [40]. NOX2 is localized in synaptic sites of neurons and plays a role in superoxide-dependent long-term potentiation and memory function [41]. Similar to previous studies, NOX2 expression increases after CIH exposure in WT mice [24]; however, a decrease of NOX2 appears in case of the deficiency of hepcidin genes. The polyunsaturated fatty acids (PUFAs) are readily be oxidized with excessive ROS [42]. 4-HNE is one of the typical lipid peroxidation products and primary attack proteins, DNA, and membrane lipids [43]. CIH exposure significantly increases 4-HNE levels, indicating that oxidative stress damage occurs in the hippocampus. Study has demonstrated that the iron level is positively correlated with NOX2 [19] and 4-HNE content, respectively [34]. These results further confirm that the CIH mice have a lower iron level and a decrease of oxidative damage.

Studies have confirmed that CIH-induced OSA could lead to neuronal apoptosis and dysfunction in the hippocampus [44] because of the production of ROS and spread of oxidative stress [45]. Apoptosis is mainly controlled by two cascades, including kinase cascades and protease cascades [46]. MAPK cascade is one of the most important members of kinase cascade and could be activated in stress responses like ROS [47]. Our previous studies showed that JNK-MAPK was activated in cardiac tissue when exposed to CIH [6]. The activated JNK declines the ratio of Bcl-2/Bax, resulting in mitochondrial dysfunction and apoptosis [19]. In our study, the activated JNK-MAPK was presented in the hippocampus of CIH mice, and the activation was suppressed in sh*Hamp* mice. Furthermore, these data suggested the correlation between JNK and iron levels during CIH exposure.

The synapse, a specialized structure of neurons, is a key part of the information transmission between neurons [48]. Synaptic plasticity is associated with learning and memory formation [49]. We observed the shortening of the active zone during CIH expose, which indicated that the synaptic transmission and plasticity were weakened [8]. BDNF has emerged as an important role in synaptic plasticity and neuronal survival [50]. On the one hand, the loss of BDNF leads to damage of long-term memory and participates in neurocognitive impairment in rodent of CIH [8, 24]. On the other hand, BDNF significantly prevents neuron damage and apoptosis induced by ROS [51]. Our results revealed that the decrease of synaptic plasticity and BDNF level were all significantly increased in the sh*Hamp* mice subjected to CIH. Therefore, these data confirm that hepcidin is involved in the dysfunction of synaptic plasticity induced by CIH.

## 5. Conclusion

In conclusion, our result demonstrates the role of hepcidin in CIH-induced cognitive impairment. First of all, hepcidin is induced during CIH exposure, accelerating the iron overload in the hippocampus and cognitive impairment. Furthermore, when the hepcidin gene is specifically knocked down in astrocyte, the excess of iron content in neuron and oxidative stress are decreased, and neuronal apoptosis and synaptic plasticity are improved when subjected to CIH.

## Data Availability

The data used to support the findings of this study are included within the article.

## Supplementary Materials

Supplementary Materials Figure S1: the structure of lentivirus plasmid LV-U6-sh*Hamp* and identification of sh*Hamp* mice. (A) The structure of lentivirus plasmid LV-U6-sh*Hamp* with the astrocytes promoters followed by *Hamp* gene shRNA sequence. (B) The expression of hepcidin mRNA by RT-PCR in the hippocampus of sh*Hamp* mice. The data are shown as the means ± SEM. ^∗^*p* < 0.05 vs. WT (*n* = 6).
