# Supplementary material for: Involvement of Hepcidin in Cognitive Damage Induced by Chronic Intermittent Hypoxia in Mice
Source: Oxid Med Cell Longev. 2021 Aug 4;2021:8520967. doi: 10.1155/2021/8520967 (PMC8357469; doi:10.1155/2021/8520967)
Supplement: Supplementary Materials — Figure S1: the structure of lentivirus plasmid LV-U6-shHamp and identification of shHamp mice. (A) The structure of lentivirus plasmid LV-U6-shHamp with the astrocytes promoters followed by Hamp gene shRNA sequence. (B) The expression of hepcidin mRNA by RT-PCR in the hippocampus of shHamp mice. The data are shown as the means ± SEM. ∗p < 0.05 vs. WT (n = 6). [file 8520967.f1.docx]

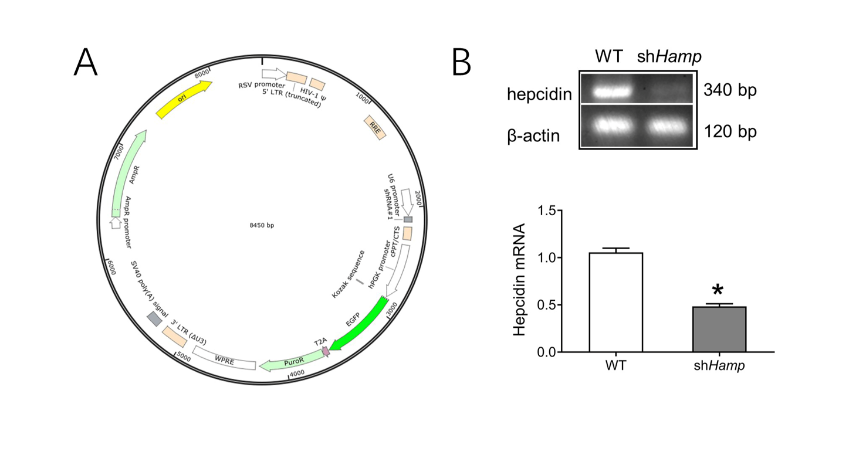


**Figure S1. The structure of lentivirus plasmid LV-U6-sh*Hamp* and identify of sh*Hamp* mice.** (A) The structure of lentivirus plasmid LV-U6-sh*Hamp* with the astrocytes promoters followed by *Hamp* gene shRNA sequence. (B) The expression of hepcidin mRNA by RT-PCR in [hippocampus](javascript:;) of sh*Hamp* mice. The data are shown as the means ± SEM. ^*^*p* < 0.05 vs. WT (n = 6).
